# Evaluation of Dental Fear and Dental Caries in Pediatric Patients with Molar Incisor Hypomineralization

**DOI:** 10.3290/j.ohpd.c_2316

**Published:** 2025-10-20

**Authors:** Hilal Özbey İpek, Arif Bolaca

**Affiliations:** a Hilal Özbey İpek Assistant Professor, Pamukkale University, Faculty of Dentistry, Department of Pediatric Dentistry, Kınıklı, Denizli, Turkey. Performed the questionnaire administration, and statistical evaluation, wrote the manuscript.; b Arif Bolaca Assistant Professor, Pamukkale University, Faculty of Dentistry, Department of Pediatric Dentistry, Kınıklı, Denizli, Turkey. Study design, performed the dental examinations, and statistical evaluation.

**Keywords:** children, dental anxiety, dental caries, dental fear, MIH, molar incisor hypomineralization.

## Abstract

**Purpose:**

Children with molar incisor hypomineralization (MIH) often experience dental hypersensitivity and discomfort during dental procedures. These factors can contribute to the dental fear and anxiety (DFA) in children with MIH. The aim of the study was to examine the relationship between MIH, dental caries, and fear.

**Materials and Methods:**

In total, 122 children (MIH group=60; Control group=62) aged 8–12 were included in the study and examined. Caries and teeth affected by MIH were recorded. After the examination, The Children’s Fear Survey Schedule-Dental Subscale (CFSS-DS) was administered to each child.

**Results:**

The mean DMFT/dmft index, total CFSS-DS score, and presence of dental fear were statistically significantly higher in the MIH group than control group. The number of children with severe MIH was statistically significantly greater than those with mild MIH. No statistically significant differences were observed between children with mild and severe MIH in terms of mean DMFT/dmft index, CFSS-DS total score, or presence of fear.

**Conclusion:**

A relationship was observed between MIH and DFA. Similarly, caries experience of children with MIH was statistically significantly higher than those without MIH.

Molar incisor hypomineralization (MIH), initially defined in Sweden, is a qualitative dental anomaly characterized by enamel that is more porous and fragile than normal enamel, making it less resistant to masticatory forces.^33^ The prevalence of this condition is reported to be between 2.4% and 40.2%, and the defects may range from distinct white-yellow opacities to areas of broken down tissue.^19,32^ MIH may affect one or more of the first permanent molars and incisors. In addition, the permanent canines and second primary molars may also be impacted.^19^ Although the exact etiology of MIH remains unknown, various studies have suggested that it may be associated with medical and genetic factors, as well as environmental pollution.^1^ MIH treatment can include fissure sealants, microabrasion, root canal treatments, composite restorations, full coronal coverage restorations, and extractions, depending on the severity of the disorder.^24^ Furthermore, restoration losses were observed due to the altered enamel structure. Studies have reported that molars affected by MIH in children require treatment approximately ten times more frequently than molars in children without the condition.^10^ Owing to the porous structure of teeth affected by MIH, bacteria can readily penetrate the enamel and dentin, potentially leading to subclinical inflammatory responses in the pulp. This complicates the achievement of effective anesthesia and may result in painful treatments.^26^ Children with MIH often experience dental hypersensitivity and exhibit discomfort from air-water spray during dental procedures. Hypersensitivity can make it difficult for dentists to manage pain.^11,19^ Thus, all these factors can contribute to the fear of dentistry in children with MIH; however, the literature on this subject remains controversial.^25^ Dental fear and anxiety (DFA) refers to the intense negative emotions individuals experience in anticipation of or during dental treatment. Although the exact cause of DFA remains unknown, the condition is often associated with previous negative experiences of dental treatment and pain. Patients with DFA often delay or avoid dental treatment, which can lead to the progression and worsening of their oral health problems over time. DFA is a common challenge encountered by pediatric dentists, making behavior management difficult and potentially influencing treatment preferences and quality.^5,14^ The present study aimed to examine the relationship between MIH, dental caries, and fear. The null hypothesis stated that MIH has no impact on the prevalence of dental caries or the presence of dental fear in children.

## MATERIALS AND METHODS

This cross-sectional study was approved by the Ethics Committee of Pamukkale University, Faculty of Medicine (No.19; 27/12/2022), and all the procedures performed in the study were conducted in accordance with the ethical standards given in the Declaration of Helsinki.

### Study Design and Sample

In the power analysis of the present study conducted with the G*power 3.1 program software (University of Kiel, Kiel, Germany), an effect size of 0.46 was derived from the reference study.^29^ Based on the sample size analysis using an alpha error probability of 0.05 and a power value of 0.80, the minimum required total sample size was calculated to be 120 (60 children per group). Informed consent was obtained from each child’s parent for the dental examination and survey administration. Children with MIH aged 8 to 12 years who visited the Pediatric Dentistry Department of Pamukkale University Faculty of Dentistry, for examination or treatment were included in the study. The Pamukkale University Faculty of Dentistry is a tertiary referral center where patients present either by self-referral or through referrals from primary care dentists. In Turkey, dental treatment in university clinics is covered by the government insurance system, ensuring that patients from varied socioeconomic backgrounds receive care free of charge. All children aged 8 to 12 years who attended our Pediatric Dentistry Clinic between January 2023 and May 2024 were screened for the presence of MIH. Eligible children were invited to participate, and those whose parents provided informed consent were enrolled consecutively. To form the comparison group, the same number of control group patients with similar age and sex distribution to the MIH patients were randomly selected during the same period. Recruitment continued until the target sample size was reached, and finally 60 children with MIH and 62 healthy controls were included. In total, 122 children (MIH group=60; Control group=62) with fully erupted permanent first molars, who were systemically healthy and had no physical or mental disabilities were included in the study. Children with fluorosis, hypoplasia, fixed orthodontic appliances, systemic diseases, and/or other syndromes were excluded from the control group of the study. Children with fluorosis, hypoplasia other than MIH, fixed orthodontic appliances, systemic diseases, and/or other syndromes were also excluded from the MIH group of the study. The data were collected between January 2023 and May 2024.

### Dental Examination

Clinical examinations of the children were performed by a trained and experienced pediatric dentist (A.B.) who had prior postgraduate training and clinical experience in the diagnosis of MIH and caries using the DMFT/dmft indices. Before the commencement of the study, the examiner underwent a calibration process that included both theoretical and practical stages. Initially, the diagnostic criteria of the European Academy of Pediatric Dentistry (EAPD) for MIH^19^ and the WHO criteria^34^ for caries were reviewed, and clinical photographs of children with MIH and other enamel defects were assessed under the supervision of a senior pediatric dentist (H.Ö.İ.) to ensure consistency in diagnosis. Following this, the examiner conducted clinical examinations of 15 children with MIH and repeated these assessments after a one-week interval. In addition, 15 children with caries were also examined twice with a one-week interval between assessments; these patients were not included in the study. The intra-examiner κ-value for MIH diagnosis was 0.92, indicating excellent agreement, and the intra-examiner κ-value for DMFT/dmft scoring was 0.98, indicating excellent reliability. Before examination, the teeth were cleaned using a standard polishing brush. Children were examined under a unit light with the help of a dental mirror and a probe. Moreover, caries was evaluated using the DMFT/dmft index approved by the World Health Organization.^34^ Teeth affected by MIH were evaluated according to the MIH Clinical Diagnostic Guide of the European Academy of Pediatric Dentistry (EAPD) and classified according to the severity of the condition (mild or severe). This index serves as a reliable and accurate diagnostic tool.^19^

### Administration of the Survey

After the examination, the Turkish version of the Children’s Fear Survey Schedule-Dental Subscale (CFSS-DS) was administered to each child by a different pediatric dentist (H.Ö.İ.). The CFSS-DS is a psychometric questionnaire used to assess DFA in children. Additionally, the reliability and validity of the scale have been demonstrated across various populations.^22^ Kuscu et al^16^ assessed the validity and reliability of the Turkish version of the CFSS-DS. The 15-item questionnaire encompasses a variety of dental stimuli, including injections, mouth opening, drilling, tooth cleaning, and other general medical procedures. Children rated their level of fear for each item on a 5-point Likert scale, where 1 indicated “not afraid at all” and 5 indicated “very afraid.” The scores on the questionnaire ranged from 15 to 75. Children with a total CFSS-DS score of 38 and above were classified as having dental fear.^16^

### Statistical Analysis

Statistical analyses were performed using Number Cruncher Statistical System 2007 Statistical Software (Kaysville, Utah, USA). Descriptive statistical methods (means, standard deviations, medians, and interquartile ranges) were used to evaluate the data. Additionally, the distribution of variables was examined using the Shapiro–Wilk normality test. An independent t-test was used to compare paired groups of variables demonstrating normal distribution, the Mann–Whitney U-test was used to compare paired groups of variables that did not exhibit normal distribution, while the chi-squared test was employed for the comparison of categorical data. The results were evaluated at a significance level of p<0.05.

## RESULTS

A total of 152 patients, aged 8 to 12 years, were initially evaluated for eligibility. Eight patients (in MIH group) and their parents chose not to participate in the study. Twenty-two patients (12 patients in MIH group; 10 patients in control group) were excluded as they did not fulfill the inclusion criteria. The final sample comprised 122 patients who met the inclusion criteria and were subsequently enrolled in the study. The study sample consisted of 48 boys (39.34%) and 74 girls (60.66%) with an average age of 9.30 ± 1.30 years.

No statistically significant differences were observed in the mean age (p=0.807) or sex distribution (p=0.822) between the control and MIH groups (Table 1).

**Table 1:** Age and gender distribution of the groups

	Control group	MIH group	p
Age	Mean±SD	9.23±1.30	9.28±1.29	0.807*
Gender n (%)	Boys	25 (40.32%)	23 (38.33%)	0.822+
Girls	37 (59.68%)	37 (61.67%)
*Independent t-test; +chi-squared test; MIH: molar incisor hypomineralization; SD: standard deviation.

The mean DMFT/dmft index (p=0.031), mean total CFSS-DS score (p=0.008), and presence of dental fear (p=0.046) were all identified to be statistically significantly higher in the MIH group than those in the control group (Table 2).

**Table 2:** Comparison of groups in terms of DMFT/dmft index, CFSS-DS total score and presence of fear

		Control group	MIH group	p
DMFT/dmft index	Mean±SD	6.85±2.84	8.03±3.09	0.031^†^
Median (IQR)	7 (4-9)	7.5 (6-11)
CFSS-DS Total score	Mean±SD	22.53±4.86	25.52±7.19	0.008*
Presence of fear n (%)	Fear (-) (CFSS-DS<38)	61 (98.39%)	54 (90.00%)	0.046^+^
Fear (+) (CFSS-DS≥38)	1 (1.61%)	6 (10.00%)
*Independent t-test; ^†^Mann-Whitney U-test; +chi squared test; CFSS-DS: Children’s Fear Survey Schedule-Dental Subscale; DMFT/dmft: decayed, missing, filled permanent/primary teeth number; IQR: inter quantile range; MIH: molar incisor hypomineralization; SD: standard deviation. Bold indicates statistical significance.

The mean CFSS-DS scores for each survey item were compared between the groups. No statistically significant differences were noted between the groups for any of the items, except for two (Table 3).

**Table 3:** Comparison of each CFSS-DS item between groups

	Control group	MIH group	p^†^
1. Dentists	1.53±0.76	1.70±0.79	0.178
2. Doctors	1.31±0.56	1.48±0.70	0.133
3. Injections	2.39±1.27	2.47±1.28	0.708
4. Having somebody examine your mouth	1.19±0.47	1.33±0.82	0.629
5. Having to open your mouth	1.16±0.45	1.20±0.51	0.721
6. Having a stranger touch you	1.84±1.18	1.93±1.16	0.572
7. Having somebody look at you	1.37±0.68	1.80±1.19	0.065
8. Dentist drilling	1.68±0.86	1.83±1.08	0.581
9.The sight of dentist drilling	1.61±0.93	1.77±1.13	0.589
10. The noise of dentist drilling	1.19±0.47	1.43±0.87	0.093
11. Having somebody put instruments in your mouth	1.39±0.75	1.72±1.03	0.039
12. Choking	2.42±1.37	2.73±1.34	0.142
13. Having to go to the hospital	1.34±0.60	1.72±1.17	0.135
14. People in white uniform	1.05±0.28	1.27±0.80	0.024
15. Having the dentist clean your teeth	1.06±0.25	1.13±0.47	0.464
^†^Mann-Whitney U-test; CFSS-DS: Children’s Fear Survey Schedule-Dental Subscale; MIH: molar ıncisor hypomineralization. Bold indicates statistical significance.

Within this study sample, the number of children with severe MIH was statistically significantly greater than those with mild MIH (p=0.0001). As this was a clinic-based sample, these findings reflect only the distribution of MIH severity among participants and should not be interpreted as epidemiological prevalence. No statistically significant differences were observed between children with mild and severe MIH in terms of mean age (p=0.846), sex distribution (p=0.524), mean DMFT/dmft index (p=0.697), mean CFSS-DS total score (p=0.554), or presence of fear (p=0.453) (Table 4).

**Table 4:** Comparison of the number, age and gender distribution, DMFT/dmft index, CFSS-DS total score and presence of fear of children with mild MIH and severe MIH

	Severity of MIH	p
Mild	Severe
Number of children n (%)		18 (30%)	42 (70%)	0.0001^+^
Age	Mean±SD	9.33±1.30	9.26±1.27	0.846*
Gender n (%)	Boys	8 (44.44%)	15 (35.71%)	0.524^+^
Girls	10 (55.56%)	27 (64.29%)
DMFT/dmft Index	Mean±SD	7.83±3.07	8.12±3.13	0.697^†^
Median (IQR)	7.5 (5.75–10.25)	8 (6-11)
CFSS-DS Total Score	Mean±SD	24.67±8.15	25.88±6.82	0.554*
Presence of Fear n (%)	Fear (-) (CFSS-DS<38)	17 (94.44%)	37 (88.10%)	0.453^+^
Fear (+) (CFSS-DS≥38)	1 (5.56%)	5 (11.90%)
*Independent t-test; †Mann-Whitney U-test; +chi-squared test; CFSS-DS: Children’s Fear Survey Schedule-Dental Subscale; DMFT/dmft: decayed, missing, filled permanent/primary teeth number; IQR: inter quantile range; MIH: molar incisor hypomineralization; SD: standard deviation. Bold indicates statistical significance.

Mean CFSS-DS scores for each survey item were compared between children with mild and severe MIH. No statistically significant differences were noted for any of the items, except for two (Table 5).

**Table 5:** Comparison of each CFSS-DS item between children with mild MIH and severe MIH

	Severity of MIH	p^†^
Mild	Severe
1. Dentists	1.89±0.68	1.62±0.83	0.093
2. Doctors	1.50±0.86	1.48±0.63	0.743
3. Injections	2.33±1.14	2.52±1.35	0.703
4. Having somebody examine your mouth	1.72±1.18	1.17±0.54	0.033
5. Having to open your mouth	1.33±0.69	1.14±0.42	0.275
6. Having a stranger touch you	1.56±0.86	2.10±1.25	0.111
7. Having somebody look at you	1.28±0.75	2.02±1.28	0.015
8. Dentist drilling	1.72±1.27	1.88±0.99	0.230
9. The sight of dentist drilling	1.61±1.20	1.83±1.10	0.280
10. The noise of dentist drilling	1.61±1.04	1.36±0.79	0.244
11. Having somebody put instruments in your mouth	1.67±1.14	1.74±0.99	0.404
12. Choking	2.28±1.13	2.93±1.39	0.081
13. Having to go to the hospital	1.78±1.44	1.69±1.05	0.613
14. People in white uniform	1.11±0.32	1.33±0.93	0.533
15. Having the dentist clean your teeth	1.28±0.75	1.07±0.26	0.245
^†^Mann-Whitney U-test; CFSS-DS: Children’s Fear Survey Schedule-Dental Subscale; MIH: molar incisor hypomineralization. Bold indicates statistical significance.

## DISCUSSION

Fear of dentistry is defined as an enhanced physiological, behavioral, and emotional response to a threatening stimulus. Negative experiences associated with dental treatment, particularly during childhood and adolescence, are linked to the development of dental fear.^14^ Teeth affected by MIH can cause discomfort in children due to hypersensitivity, and their treatment may be painful because of challenges in achieving effective anesthesia.^19,26^ This study aimed to determine whether children with MIH experience more DFA and caries compared to healthy children.

In studies assessing DFA, various scales, including the Dental Anxiety Questionnaire, the Dental Visit Satisfaction Scale, and the Modified Child Dental Anxiety Scale, were employed.^12^ Although it is discussed that the cut-off value of the CFSS-DS should be adjusted based on age and sex,^18^ it remains the most widely utilized instrument for assessing dental fear across diverse populations.^14^ Therefore, the CFSS-DS was used to evaluate the DFA in our study. In the literature, the diagnosis of MIH was primarily based on the EAPD index, along with the Developmental Defects of Enamel Index.^12^ In the present study, a specific and validated EAPD index was used to diagnose MIH.

In the present study, the CFSS-DS total score and presence of fear were statistically significantly high in children with MIH. Sajadi et al^28^ reported in 2022 that the presence of dental fear in children with MIH was 46 times higher than in those without MIH. Other studies have reported that DFA is relatively common in children with this condition.^11,27^ In a recently published study that aimed to determine dental fear by measuring children’s cortisol levels, the levels were statistically significantly higher in children with MIH than in those without.^6^ In contrast, while some studies did not observe a statistically significant association between DFA and MIH in children,^12,22,23^ some others reported an increase in DFA among children with MIH, although the findings were not statistically significant.^6,24,25,26,27^ However, some researchers have reported that children with MIH may experience behavioral management challenges and compromised oral health-related quality of life, even if DFA is not observed.^29,30,31^ Maladaptive behavior and DFA in children are not the same concepts. Even if a child does not experience a DFA, they may still exhibit uncooperative behavior during treatment.^11^ Implementation of behavioral guidance techniques in children with MIH is crucial. In addition, if factors that may contribute to DFA, such as hypersensitivity or inadequate anesthesia, occur during treatment, they should be documented by the dentist. Thus, dentist should remain cautious about reducing pain in subsequent sessions and preventing the child from being afraid. If a child experiences persistent pain during treatment, it is likely to lead to the development of fear and the emergence of maladaptive behaviors.

The findings of a few studies investigating the relationship between MIH severity and DFA differ. Sajadi et al^28^ and Özükoç et al^21^ reported that dental fear increases with the severity of MIH, noting that the frequency of dental fear is significantly higher in children with severe defects compared to those with mild forms of the condition. Additionally, Contac et al^6^ reported that cortisol levels in children increased as the severity of MIH increased. However, in the present study, although the difference was not statistically significant, children with severe MIH had slightly more dental fear than those with mild MIH. These findings should be interpreted with caution, as the study was not powered for subgroup analyses and the mild MIH group was considerably smaller than the severe group, limiting the reliability of such comparisons. Similarly, Kosma et al^15^ reported that the relationship between CFSS-DS scores and DFA in children with MIH was not statistically significant in terms of disorder severity. The outcomes of other studies also support these results.^17,29^ One possible explanation for these findings is that children with MIH may have become accustomed to the dental environment due to the frequency of their dental visits. They may have developed the perception that the treatment would alleviate their pain. In addition, behavioral management techniques may be used more intensively by dentists due to the increased awareness of MIH and the challenges associated with anesthetizing affected teeth.

In the study conducted by Sezer et al,^29^ “injections” were the most feared item in the groups with and without MIH. Statistically significant differences between the two groups were observed for the items “having somebody look at you” and “the sight of the dentist drilling”. In the present study, the most commonly feared items were “choking” and “injections”. Although children with MIH exhibited higher fear levels than those without MIH across all items, only the items “having somebody put instruments in your mouth” and “people in a white uniform” demonstrated statistically significant differences. While the “injections” item ranks second in fearfulness among healthy children and those with MIH overall, it is the most feared item among children with mild and severe MIH. This result may demonstrate the pain and fear experienced by children with MIH during anesthesia.

In the present study, patients with MIH demonstrated higher caries index scores compared to healthy patients. This result is similar to those of many previous studies.^4,8,13,15,23,30^ Only one study reported that the number of carious lesions was not associated with the presence of MIH.^9^ In addition, in our study, MIH severity and caries index scores were evaluated, and no differences were noted between mild and severe MIH. The patients included in the study were selected from among those with MIH who visited the university clinic and were under follow up. The reason why there was no difference between the DMFT/dmft index scores of patients with severe and mild MIH may be that all patients diagnosed with MIH were given preventive treatments (fissure sealant, fluoride treatment) and received education on oral hygiene and nutrition at the initial appointment. However, Kosma et al^15^ and Ghanim et al^7^ reported that children with severe MIH had higher caries scores than those with mild MIH. These different findings may be because the aforementioned studies were conducted in a large population.

Based on these results, the null hypothesis was rejected. However, some limitations should be considered when evaluating the results of this study. Patients’ past dental experiences and the social and psychological characteristics of their families may have had an impact on DFA. Additionally, while administering the questionnaires to the patients, it was not recorded whether they had received dental treatment before or whether it was their first dental appointment. The effects of these variables were not evaluated in this study. Furthermore, patients were evaluated in terms of the number of decayed, extracted, and filled teeth, but not on the number of affected surfaces. As teeth with MIH may involve multiple surfaces, it would have been logical to compare healthy individuals and patients with MIH. In addition, differences between ages or genders could have been evaluated in terms of DFA. Furthermore, comparisons of behavioral problems between the groups could have been conducted. An additional limitation is that the sample size calculation was performed for overall group comparisons (MIH vs control) rather than for subgroup analyses of mild and severe MIH. Moreover, the smaller size of the mild MIH group further reduced the reliability of these subgroup comparisons. Therefore, the results can not be generalized to population-level epidemiology, as the DFA of children with MIH may vary across different cultures and countries, and even across different parts of the country. Accordingly, in the future, studies with more samples should be conducted which address these limitations.

## CONCLUSION

A relationship was observed between MIH and DFA. Children with MIH showed higher CFSS-DS scores than children without MIH. Similarly, a statistically significant association was observed between MIH and caries experience. The mean DMFT/dmft index was higher in the MIH group than those in the control group.
